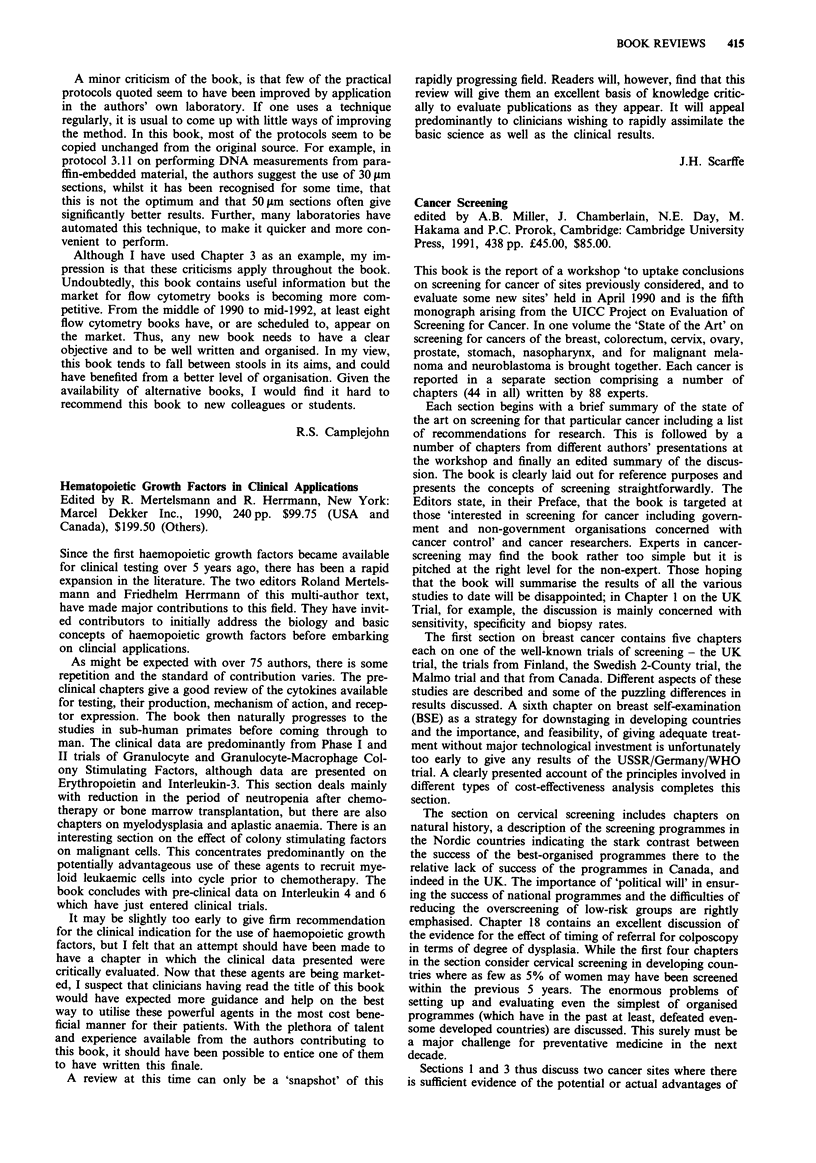# Hematopoietic Growth Factors in Clinical Applications

**Published:** 1992-08

**Authors:** J. H. Scarffe


**Hematopoietic Growth Factors in Clinical Applications**
Edited by R. Mertelsmann and R. Herrmann, New York: Marcel Dekker Inc., 1990, 240 pp. $99.75 (USA and Canada), $199.50 (Others).

Since the first haemopoietic growth factors became available for clinical testing over 5 years ago, there has been a rapid expansion in the literature. The two editors Roland Mertelsmann and Friedhelm Herrmann of this multi-author text, have made major contributions to this field. They have invited contributors to initially address the biology and basic concepts of haemopoietic growth factors before embarking on clincial applications.

As might be expected with over 75 authors, there is some repetition and the standard of contribution varies. The pre-clinical chapters give a good review of the cytokines available for testing, their production, mechanism of action, and receptor expression. The book then naturally progresses to the studies in sub-human primates before coming through to man. The clinical data are predominantly from Phase I and II trials of Granulocyte and Granulocyte-Macrophage Colony Stimulating Factors, although data are presented on Erythropoietin and Interleukin-3. This section deals mainly with reduction in the period of neutropenia after chemotherapy or bone marrow transplantation, but there are also chapters on myelodysplasia and aplastic anaemia. There is an interesting section on the effect of colony stimulating factors on malignant cells. This concentrates predominantly on the potentially advantageous use of these agents to recruit myeloid leukaemic cells into cycle prior to chemotherapy. The book concludes with pre-clinical data on Interleukin 4 and 6 which have just entered clinical trials.

It may be slightly too early to give firm recommendation for the clinical indication for the use of haemopoietic growth factors, but I felt that an attempt should have been made to have a chapter in which the clinical data presented were critically evaluated. Now that these agents are being marketed, I suspect that clinicians having read the title of this book would have expected more guidance and help on the best way to utilise these powerful agents in the most cost beneficial manner for their patients. With the plethora of talent and experience available from the authors contributing to this book, it should have been possible to entice one of them to have written this finale.

A review at this time can only be a 'snapshot' of this rapidly progressing field. Readers will, however, find that this review will give them an excellent basis of knowledge critically to evaluate publications as they appear. It will appeal predominantly to clinicians wishing to rapidly assimilate the basic science as well as the clinical results.

J.H. Scarffe